# Multi‐domain cognitive resilience to Alzheimer's Disease, Lewy Body and TDP‐43 neuropathologies

**DOI:** 10.1002/alz70855_096284

**Published:** 2025-12-23

**Authors:** Tain Luquez, Dhwani Sreenivas, David A. A. Bennett, Philip L. De Jager, Vilas Menon

**Affiliations:** ^1^ Columbia University Irving Medical Center, New York, NY, USA; ^2^ Columbia University, New York, NY, USA; ^3^ Rush University, Chicago, IL, USA; ^4^ Columbia University Irving Medical Center and the Taub Institute for Research on Alzheimer's Disease and the Aging Brain, New York, NY, USA

## Abstract

**Background:**

To identify cell type‐specific transcriptional signatures of cognitive resilience against neuropathologies in Alzheimer's Disease, Lewy Body and TDP‐43.

**Methods:**

We used publicly available single nucleus RNA‐seq from the dorsolateral prefrontal cortex of 424 individuals to model the relationship between cell type‐specific gene expression and cognitive decline in specific domains (i.e., semantic, episodic, working memory, perceptual speed and visuospatial processing) in donors with and without postmortem amyloid beta, hyperphosphorylated tau, Lewy bodies and TDP‐43 using linear regression.

**Results:**

We identified that resilient donors had higher proportions of superficial layer 2/3 intratelecephalic excitatory neurons and lower relative proportions of deep layers 5/6 near‐projecting and layer 6b excitatory neurons across all neuropathologies. In addition, layer 2/3 intratelencephalic and 6b populations showed the highest number of differentially expressed genes (66 and 29 genes, respectively) in donors exhibiting resilience to Alzheimer's neuropathologies. This includes higher expression of phosphodiesterases like PDE10A, neurotransmitter modulators like RPH3A and injury response genes like GALR1. In contrast, we observe different genes involved in cognitive preservation for specific domains. For instance, Layer 6b cells have higher expression of neurite and axon growth promoters ARHGAP20 and LIMK1, respectively, in donors who retain their global cognition despite having substantial AD pathology, while those who retain their semantic memory have higher expression of MED25 and SDK1, which are involved in synaptic connectivity. Finally, we found fewer differentially expressed genes in donors who maintained their cognitive abilities in the face of Lewy body pathology (21 genes) and TDP‐43 pathology (130 genes) across all domains and cell types compared to Alzheimer's neuropathologies (660 genes total), with minimal overlap across these diseases, suggesting that the mechanisms linked to cognitive resilience may be pathology‐specific.

**Conclusions:**

Cognitive resilience is associated with higher relative proportions of superficial excitatory cortical neurons and associated transcriptional programs that promote neurotrophic and adaptive responses. The gene expression patterns associated with resilience, however, differ across specific neuropathologies and cognitive domains. These findings provide insights into the cellular and molecular underpinnings of cognitive resilience and inform potential therapeutic targets for mitigating cognitive decline across diverse neuropathologies.

